# Gene Expression in Urinary Sediment Cells as an Indicator of the Contribution of Plasma Lipids to Diabetic Kidney Disease

**DOI:** 10.1155/jdr/2349928

**Published:** 2025-10-07

**Authors:** Thaina Tavolaro Zocchio, Aritania Souza Santos, Ana Mercedes Cavaleiro, Luiz Henrique Gomes Matheus, Monique de Fátima Mello Santana, Eduarda Palanca, Ariana Tito Rodrigues, Milena Gomes Vancini, Márcia Nery, Marisa Passarelli, Maria Lúcia Corrêa-Giannella

**Affiliations:** ^1^Laboratory of Carbohydrates and Radioimmunoassays (LIM-18) From Clinical Hospital (HCFMUSP), Medical School, University of São Paulo, São Paulo, São Paulo, Brazil; ^2^Lipids Laboratory (LIM-10) From HCFMUSP, Medical School, University of São Paulo, São Paulo, São Paulo, Brazil; ^3^Program of Post-Graduation in Medicine, Nove de Julho University (UNINOVE), São Paulo, São Paulo, Brazil; ^4^Division of Endocrinology From HCFMUSP, Medical School, University of São Paulo, São Paulo, São Paulo, Brazil

**Keywords:** CD36, cubilin, FABP1, free fatty acids, interleukin 1 beta, interleukin 18, megalin, transforming growth factor beta, triglycerides, Type 1 diabetes mellitus

## Abstract

**Background:**

In individuals with Type 1 diabetes mellitus (T1D) who maintain good glycemic control and are free of chronic complications, lipid profiles are generally within normal ranges. However, impaired renal function or albuminuria alters this profile, contributing to the progression of diabetic kidney disease (DKD). This study hypothesized that, in urinary sediment cells from T1D individuals (*n* = 87), the mRNA expression of genes related to free fatty acid (FFA) uptake (*CD36*, *FABP1*, *SCL27A1*, *SLC27A2*, and *SLC27A4*, which encode *FATP1*, *FATP2*, and *FATP4*, respectively), albumin uptake (*LRP2* and *CUBN*), inflammation (*IL1B* and *IL18*), and fibrosis (*TGFB1*) would vary depending on the degree of renal function decline and of urinary albumin excretion (UAE) and correlate with each other and with plasma lipid concentrations.

**Results:**

*CD36* expression was higher in urinary sediment cells of individuals with relevant renal function decline compared to those without relevant decline. *CD36*, *IL1B*, *TGFB1*, and *SLC27A4* (at the limit of statistical significance) expressions were higher in individuals with UAE > 11 mg/g versus UAE ≤ 11 mg/g creatinine (cohort median value). *CD36* expression positively correlated with *IL1B* (*r* = 0.46) and *TGFB1* (*r* = 0.45) expressions, and *LRP2* expression positively correlated with *IL18* (*r* = 0.44) and *TGFB1* (*r* = 0.30). Expression of *SLC27A* genes also correlated with inflammatory and profibrotic genes. Plasma FFA concentrations positively correlated with *CD36* (*r* = 0.27) and *IL1B* (*r* = 0.30) expressions, while plasma triglyceride (TG) concentrations positively correlated with *CD36* (*r* = 0.22) and negatively correlated with *FABP1* (*r* = −0.28) expressions. Urinary sediment gene expression was modulated by statin (*IL18*) and angiotensin II receptor blockers (*LRP2*, *CUBN*, and *FABP1*) use.

**Conclusions:**

The expression of lipid metabolism–related genes in urinary sediment cells, correlated with proinflammatory and profibrotic genes, as well as with plasma FFA and TG, and associated with clinical indicators of renal function, provides indirect evidence for the involvement of lipids in the pathogenesis of DKD.

## 1. Background

Diabetic kidney disease (DKD) is a major complication of Type 1 diabetes mellitus (T1D). Although individuals with T1D who maintain good glycemic control often exhibit normal lipid profiles, the presence of albuminuria or impaired renal function is associated with detrimental alterations in lipid metabolism that accelerate DKD progression [1, 2]. Epidemiological cohorts such as the Diabetes Control and Complications Trial/Epidemiology of Diabetes Interventions and Complications (DCCT/EDIC) and the Finnish Diabetic Nephropathy Study (FinnDiane) have consistently shown associations between triglyceride (TG) concentrations, altered lipoprotein composition, and increased risk of DKD [2–4].

Experimental and clinical evidence suggests that disturbances in lipid handling within the kidney may drive tubular injury and fibrosis. Free fatty acids (FFAs), usually bound to albumin, are delivered into renal cells through specific transporters such as CD36 and fatty acid transport proteins (FATPs), or via the megalin (LRP2)/cubilin (CUBN) complex [5, 6]. Excessive intracellular accumulation of FFA promotes lipotoxicity, oxidative stress, and release of proinflammatory and profibrotic mediators, with deleterious effects on both tubular cells and podocytes [6–8]. FABP1, a fatty acid–binding protein expressed in proximal tubules, has been proposed as a marker of tubular injury, although its predictive value for DKD progression remains limited [9–11].

Despite these insights, the mechanisms by which dyslipidemia contributes to DKD progression remain incompletely understood. Most studies have relied on animal models or invasive kidney biopsies, restricting investigation in large cohorts. Urinary sediment cells provide a noninvasive alternative for exploring gene expression patterns related to renal injury [12, 13] and may offer new perspectives on the pathways linking lipid metabolism to DKD.

Based on this rationale, we hypothesized that, in urinary sediment cells from individuals with T1D, the expression of genes involved in FFA uptake (*CD36*, *FABP1*, *SCL27A1*, *SLC27A2*, and S*LC27A4*, which encode *FATP1*, *FATP2*, and *FATP4*, respectively), albumin uptake (*LRP2* and *CUBN*), inflammation (*IL1B* and *IL18*), and fibrosis (*TGFB1)* would vary according to renal function decline and urinary albumin excretion (UAE) and correlate with each other and with plasma lipid concentrations.

## 2. Participants and Methods

This study was conducted in accordance with the Institutional Ethics Committee and the Declaration of Helsinki. Informed consent was obtained from all participants. A total of 127 T1D individuals were recruited in the diabetes outpatient clinic at a tertiary university hospital from May 2022 until July 2024. This prospective recruitment was combined with a retrospective review of historical data from institutional records to enable longitudinal assessment of renal function decline. Individuals on renal replacement therapy, kidney transplant recipients, or individuals with loss of renal function secondary to conditions other than diabetes were not included.

At inclusion (last evaluation), demographic, clinical, and biochemical data were collected, along with fasting blood for assessment of plasma lipid profile and urine samples. Historical data from the first available outpatient record at the institution (median follow-up of 13 [interquartile range: 6–18] years) were also retrieved, including HbA1c and eGFR values (calculated by CKD-EPI [14]). Urinary albumin-to-creatinine ratio was measured in spot urine samples using nephelometry, and participants were classified as A1 (<3 0 mg/g creatinine), A2 (30–299 mg/g creatinine), or A3 (≥ 300 mg/g creatinine). According to eGFR, individuals were classified into the following categories: G1: ≥ 90; G2: 60–90; G3: 30–59; G4: 15–29, and G5: < 15 mL/min/1.73 m^2^ [15].

To calculate the rate of renal function decline, the difference between the last and the first eGFR was divided by the number of years of follow-up. Participants with an annual eGFR decline ≥ 3.5 mL/min/1.73 m^2^ were classified as decliners, whereas those with a decline < 3.5 mL/min/1.73 m^2^ were classified as nondecliners [16].

Arterial hypertension was defined as blood pressure ≥ 130/80 mmHg [17]. HbA1c was measured using high-performance liquid chromatography, while the other laboratory variables were assessed through standard laboratory techniques.

Urine samples were collected from a group of 27 individuals without diabetes and not receiving angiotensin converting enzyme inhibitor (ACEI), angiotensin II receptor blocker (ARB), or statin for gene expression analysis (control group).

### 2.1. Urinary mRNA Expression

Urine samples were collected in sterile, RNase-free 200 mL conical flasks (Nalge Nunc, Rochester, United States), kept on ice, and processed quickly. The samples were centrifuged at 3,000 × g for 30 min at 4°C, and the supernatant was carefully discarded. The cell pellet was lysed with 1 mL of TRIzol reagent (Thermo Fisher Scientific, Carlsbad, United States) and supplemented with 1 *μ*L of glycogen (20 mg/mL) to improve RNA recovery. After brief vortexing (15 s) and incubation at room temperature (5 min), 200 *μ*L of chloroform was added. The mixture was vortexed again for 15 s, allowed to stand for another 5 min, and centrifuged at 10,000 × g for 15 min at 4°C. The aqueous phase was transferred to a clean 1.5 mL tube, mixed with an equal volume of isopropanol and 100 *μ*L of 3 M sodium acetate (pH 5.2), gently inverted four times, and incubated on ice for 30 min. Following centrifugation (10,000 × g, 20 min, 4°C), the supernatant was discarded, and the pellet was washed twice with 70% ethanol, each followed by centrifugation at 10,000 × g for 10 min at 4°C. The RNA pellet was carefully air-dried by inverting the tube and removing residual liquid and then resuspended in 35 *μ*L of RNase-free water. RNA integrity and concentration were assessed using the 2100 Bioanalyzer capillary electrophoresis system (Agilent Technologies, Santa Clara, United States) [18]. Only samples showing clear 28S and 18S ribosomal bands and a minimum RNA concentration of 14 ng/*μ*L (Supporting Information 2: Figure S1) were included in the analyses (*n* = 87 in the T1D group and *n* = 16 in the control group). Reverse transcription was carried out using 200 ng of total RNA and the High-Capacity cDNA Reverse Transcription Kit (Life Technologies, Carlsbad, United States). Commercially available TaqMan probes and primers (assay IDs listed in Supporting Information 1: Table S1) were employed to evaluate the expression of the following genes by qPCR using the StepOne plus Real-Time PCR System (Life Technologies): *CD36*, *FABP1*, *SCL27A1* (encodes for FATP1), *SLC27A2* (encodes for FATP2), *SLC27A4* (encodes for FATP4), *LRP2* (encodes for megalin), *CUBN* (encodes for cubilin), *IL18*, *IL1B*, and *TGFB1*. E74-like factor 1 (*ELF1*) was selected as the reference gene based on prior validation by our group in urinary sediment cells from individuals with T1D, where it was identified as the most stable gene among 32 candidates [12]. All samples were run in duplicate. The abundance of the target mRNA was determined using the 2^−ΔCt^ method.

### 2.2. Assessment of Plasma Lipid Profile

Venous blood (20 mL) was collected after an 8-h fast. Plasma was obtained by centrifugation at 3,000 rpm at 4°C and stored at −80°C until analyses of TC, TG, and HDL cholesterol (HDL-c), the latter after apoB precipitation (Labtest do Brasil). LDL cholesterol (LDL-c) was calculated using the Friedewald formula [19]. For FFA determination (Randox NEFA Assay, Randox do Brasil Ltda.), 100 *μ*L of plasma was stored in the presence of orlistat dissolved in dimethyl sulfoxide (10 mg in 10 mL).

### 2.3. Statistical Analyses

Owing to the nonnormal distribution of the data, nonparametric tests were employed. Continuous variables are presented as median and interquartile range, and differences between groups were evaluated using the Mann–Whitney test. Categorical variables are expressed as percentages, with differences between groups evaluated by Pearson's chi-square test. Spearman's rank correlation coefficient was employed for the correlation analyses. Statistical analyses were performed by JMP software Version 8.0 (SAS Institute, Cary, United States). A *p* value of < 0.05 was considered statistically significant. This study was exploratory in nature, and a convenience sample was used.

## 3. Results

The cohort included 87 T1D individuals with urinary sediment RNA of acceptable quality and quantity, of whom 78% were women, with a median age of 38 (interquartile range: 26–48) years and diabetes duration of 22 (16–34) years at the last evaluation. The HbA1c at the first and the last evaluations was 83 (69–97) and 69 (60–81) mmol/mol, respectively. The eGFR at the first and the last evaluations was 118 (105–133) and 102 (78–123) mL/min/1.73 m^2^, respectively. At the last evaluation, 69.7% were classified as A1, 18.6% as A2, and 11.7% as A3; 64.4% were classified as G1, 17.2% as G2, 13.8% as G3, and 4.6% as G4; 23% had arterial hypertension, 28.7% were prescribed an ACEI, 19.5% were prescribed an ARB, and 50.5% were using a statin. The demographic, clinical, and biochemical characteristics of the T1D individuals according to the status of renal function decline and of UAE are shown in Table 1. When compared to those classified as nondecliners (experienced an eGFR decline < 3.5 mL/min/1.73 m^2^/year), the decliners (experienced an eGFR decline ≥ 3.5 mL/min/1.73 m^2^/year) presented lower values of BMI (approaching statistical significance) and higher values of the last HbA1c and of plasma concentrations of TGs (approaching statistical significance), shorter interval between the first and last evaluations, lower eGFR at the last evaluation, higher values of UAE, higher proportion of individuals with UAE classified as A2+A3 and higher proportion of individuals using ARB.

When compared to those presenting UAE ≤ 11 mg/g creatinine (cohort median value), the individuals with UAE > 11 mg/g creatinine presented higher values of the last HbA1c and of plasma concentrations of TGs and of FFA (approaching statistical significance), higher values of UAE, a higher proportion of individuals with UAE classified as A2+A3, and a higher proportion of individuals with arterial hypertension.

The control group included 16 individuals without diabetes mellitus with urinary sediment RNA of acceptable quality and quantity, of whom 68.8% were women, with a median age of 40 (29–52.5) years and a median BMI of 24 (22.2–28.7) kg/m^2^. The only clinical condition reported in this group was hypertension (12.5%) (Supporting Information 1: Table S2), and none of the individuals showed evidence of renal function decline.

### 3.1. Gene Expression in Urinary Sediment Cells

**Figure 1:**
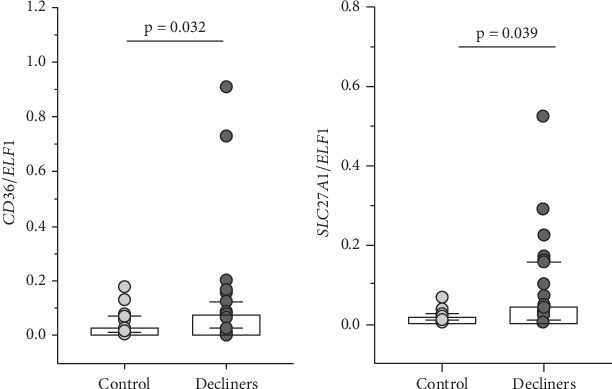
Relative expression of (a) *CD36* and of (b) *SLC27A1* in urinary sediment cells from individuals without diabetes mellitus (control group) and from Type 1 diabetes individuals presenting an estimated glomerular filtration rate decline ≥ 3.5 mL/min/1.73 m^2^/year (decliners). The horizontal line within each box plot represents the median, the box plot limits refer to the interquartile range (25th to 75th percentiles), and the bars represent the 10th and 90th percentiles.

Comparisons between the T1D group and the control group (nondiabetic) showed that only *SLC27A1* expression was significantly higher in the T1D group (*p* = 0.017), while *SLC27A2* expression was higher at the limit of statistical significance (*p* = 0.074) (Supporting Information 3: Figure S2). In addition, when comparing the control group with the subgroup of T1D individuals with the worst clinical outcome (decliners), higher expressions of *CD36* (*p* = 0.032) and of *SLC27A1* (*p* = 0.039) were observed in decliners (Figure 1).

No significant differences in the expression of any evaluated genes were detected when comparing T1D individuals stratified by UAE (A1 vs. A2+A3) or by eGFR categories (G1+G2 vs. G3+G4) (data not shown).

**Figure 2:**
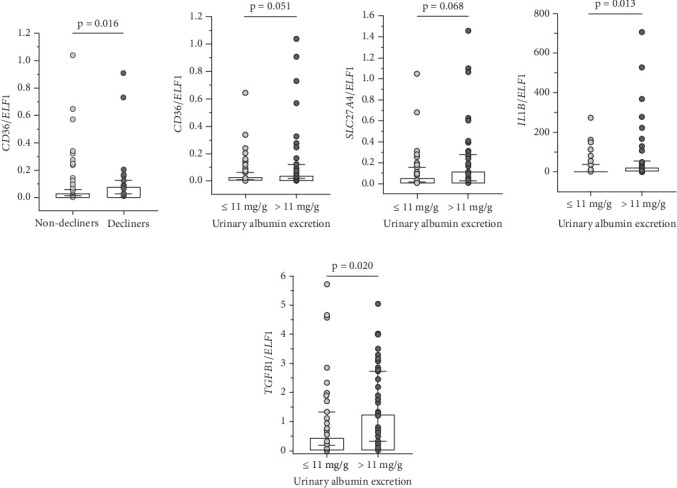
Relative expression of (a, b) *CD36*, (c) *SLC27A4*, (d) *IL1B*, and (e) *TGFB1* in urinary sediment cells from Type 1 diabetes individuals stratified by renal function decline or by urinary albumin excretion. Nondecliners: estimated glomerular filtration rate (eGFR) decline < 3.5 mL/min/1.73 m^2^/year; decliners: eGFR decline ≥ 3.5 mL/min/1.73 m^2^/year. The horizontal line within each box plot represents the median, the box plot limits refer to the interquartile range (25th to 75th percentiles), and the bars represent the 10th and 90th percentiles.

*CD36* expression was significantly higher in those T1D individuals classified as decliners in comparison to nondecliners (*p* = 0.016; Figure 2a). In addition, expressions of *CD36* (*p* = 0.051), *SLC27A4* (*p* = 0.068), *IL1B* (*p* = 0.013), and *TGFB1* (*p* = 0.020) were higher in individuals with UAE > 11 mg/g creatinine compared to those with UAE ≤ 11 mg/g creatinine (Figures 2b, 2c, 2d, and 2e).

**Figure 3:**
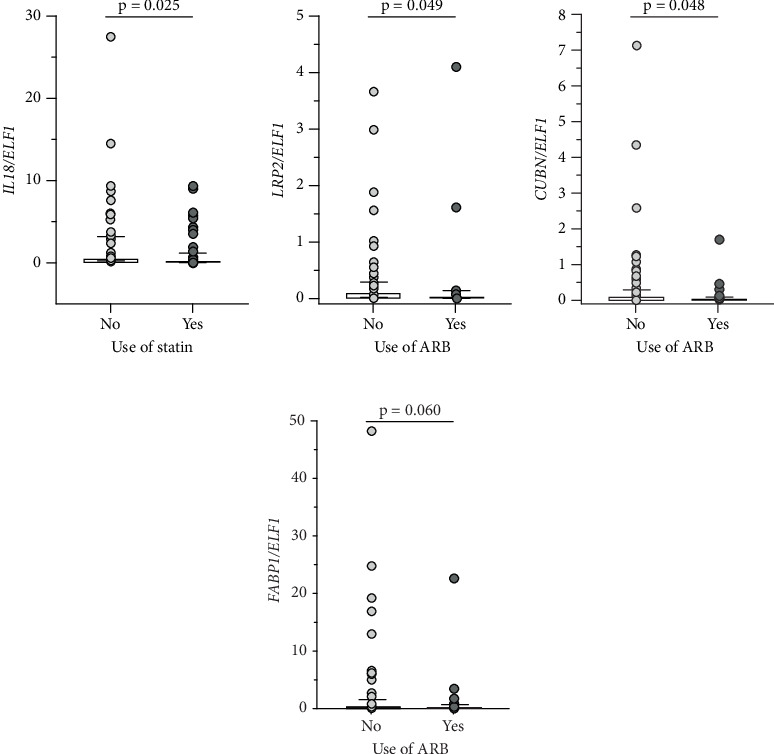
Relative expression of (a) *IL18*, (b) *LRP2*, (c) *CUBN*, and (d) *FABP1* in urinary sediment cells from Type 1 diabetes individuals prescribed or not prescribed statins and angiotensin II receptor blockers (ARB). The horizontal line within each box plot represents the median, the box plot limits refer to the interquartile range (25th to 75th percentiles), and the bars represent the 10th and 90th percentiles.

We also examined whether gene expression was influenced by medication use. T1D individuals on statin therapy showed lower *IL18* expression (*p* = 0.025; Figure 3a) compared to those not on statins. Similarly, those using ARB exhibited lower expressions of *LRP2* (*p* = 0.049), *CUBN* (*p* = 0.048), and *FABP1* (*p* = 0.060) compared to nonusers (Figures 3b, 3c, and 3d).

Gene expression correlations in the T1D group and in the control group are shown in Table 2, while Table 3 presents the correlations among gene expression and other variables within the T1D group.

## 4. Discussion

In the present study, the analysis of urinary sediment cells from T1D individuals revealed differential expression of mRNA related to FFA uptake (*CD36* and *SLC27A4*), inflammation (*IL1B*), and fibrosis (*TGFB1*) according to different degrees of renal function decline and of UAE. Correlations were found between gene expressions and plasma lipid concentrations, providing indirect evidence for lipid involvement in the pathogenesis of DKD.

Studying gene expression in human urinary sediment is a useful strategy for investigating mechanisms potentially involved in the pathogenesis of nephropathies, as the sediment contains shed renal cells. However, a key challenge is obtaining high-quality RNA from cells stored in urine, a hostile environment with a low pH and conditions favorable for RNase activity [18]. In a previous study, we characterized the expressions of *SGLT2* and *NPHS2* (podocin), as markers of proximal tubule cells and podocytes, respectively [12]. Therefore, the discussion of this study's findings primarily focuses on these two cell types, which are represented in the urinary sediment.

When comparing gene expression between the T1D and control groups, only *SLC27A1* and *SLC27A2* (at the limit of statistical significance) mRNA levels were higher in the T1D group. FATP1 and FATP2, respectively, encoded by these genes, along with FATP4 (encoded by *SLC27A4*), are key transporters of FFA in the renal tubules, and their increased activity is believed to contribute to tubular FFA overload in DKD [20]. It is interesting to note that, except for *TGFB1*, mRNA content was notably homogeneous (and low) in the control group, while in the T1D group, it was highly heterogeneous, with some individuals exhibiting elevated expression levels. To explore whether this heterogeneity was associated with more advanced renal involvement, we compared the control group with T1D individuals who had the worst renal outcome (decliners). In this analysis, only *CD36* and *SLC27A1* expressions were higher in the decliner group.

When T1D individuals were stratified by the rate of renal function decline, the group classified as decliners exhibited poorer glycemic control in the most recent evaluation, higher plasma TG concentrations (approaching statistical significance), and a shorter follow-up period compared to the nondecliner group. This latter finding may reflect the higher proportion of individuals with hyperfiltration (eGFR > 130 mL/min/1.73 m^2^ in women and > 140 mL/min/1.73 m^2^ in men) in the decliner group (33%) compared to the nondecliner group (21.6%), as hyperfiltrating individuals may experience more rapid declines in renal function over a shorter observation period [21]. Gene expression analysis revealed significantly higher *CD36* expression in the decliner group. Although the proportion of individuals with UAE categories A2 and A3 was greater in the decliner group, when A2 and A3 individuals were pooled and compared to A1 individuals (irrespective of the rate of renal function decline), no significant differences were observed in the expression of any of the evaluated genes.

Given the exploratory nature of this study, we also evaluated T1D individuals stratified according to UAE values either ≤ or > 11 mg/g creatinine (median of the T1D group). The group with excretion greater than the median showed significantly higher expression of *CD36*, *SLC27A4* (at the limit of statistical significance), *IL1B*, and *TGFB1*. It is noteworthy that both in the stratification by renal function decline rate and by UAE median, the nondecliner and UAE ≤ median groups exhibited similar albuminuria values (9 and 5 mg/g creatinine, respectively), as did the decliner and UAE > median groups (54 and 42.5 mg/g creatinine, respectively). Although these two stratification criteria did not correspond to the same individuals, they shared comparable median UAE values. In the stratification by UAE, however, the group with the worse phenotype exhibited higher TG and FFA concentrations. Therefore, we speculate that the more pronounced changes in gene expression observed with this stratification may reflect the presence of two groups with distinct TG and FFA profiles.

Another possible explanation for the observed differences in *CD36*, *SLC27A4*, *IL1B*, and *TGFB1* expression when individuals were stratified by UAE values ≤ or > 11 mg/g of creatinine (still within the A1 range), but not when category A1 was compared to categories A2+A3, is that changes in the expression of these genes may begin at a low threshold of albuminuria.

CD36 has been implicated in DKD, where its upregulation in response to high glucose concentrations promotes apoptosis in proximal tubular cells [22]. Inhibition of CD36 expression has been shown to suppress albumin-induced TGF beta elevation in renal tubular cells [23]. In a lupus nephritis model, CD36 contributed to podocyte injury, with a positive correlation observed between *CD36* expression and markers of NLRP3 inflammasome activation, including *IL1B* [24]. The increased expressions of *CD36*, *IL1B*, and *TGFB1* in the group with UAE > 11 mg/g of creatinine, along with moderate positive correlations between expressions of *CD36* with *IL1B* and with *TGFB1* exclusively in the T1D group, corroborate the involvement of pathways previously implicated in kidney diseases in preclinical models [23, 25]. Moreover, the positive correlations between plasma FFA and TG concentrations and *CD3*6 expression, and between FFA concentrations and *IL1B* expression, further support the detrimental impact of dyslipidemia on the progression of DKD.

FATP4 has also been implicated in DKD; in *db/db* mice, increased renal expression of *Slc27a4* mRNA and FATP4 protein in glomeruli correlated strongly with albuminuria [26]. These data provide biological plausibility for our findings in urinary sediment cells. Further support for the involvement of FATPs in DKD comes from the correlations observed between their expression and *TGFB1*, *IL1B*, and *IL18* exclusively in the T1D group.

The analysis of statin use revealed a significant reduction in *IL18* expression in the urinary sediment of individuals using this drug class, an effect that could be related to inhibition of the canonical NLRP3 inflammasome pathway, which would decrease caspase-1 activation and reduce IL18 production, subsequently lowering the need for its mRNA synthesis. Inhibition of this pathway, with reductions in both *IL18* and *IL1B* expression, has been previously demonstrated in human vascular endothelial cells exposed to atorvastatin in a dose- and time-dependent manner, independent of its cholesterol-lowering effects [27]. Additionally, atorvastatin has been shown to inhibit NLRP3 inflammasome activation induced by calcium oxalate crystals in cultured renal tubular cells, reducing concentrations of IL18, IL1 beta, IL6, and tumor necrosis factor (TNF) in the culture medium [28]. Our findings suggest that statins exert pleiotropic effects on the kidneys. Notably, statin use did not decrease *IL1B* expression, even though its production is also stimulated by the NLRP3 inflammasome, indicating that these two cytokines may be regulated by distinct pathways. Further supporting this, the correlation between *IL18* and *IL1B* expression was only moderate in the T1D group and nonsignificant in the Control group.

The analysis of ARB use revealed a significant reduction in the expression of *LRP2*, *CUBN*, and *FABP1* in urinary sediment of individuals using this drug class. These three genes exhibited strong or very strong correlations with each other in both T1D and control groups, and their expression was consistently modulated by ARB, suggesting they may be regulated by shared control mechanisms. A potential common regulatory mechanism for these genes is the peroxisome proliferator-activated receptor (PPAR) family. PPAR-responsive elements have been identified in the promoter region of the human *LRP2* gene, and PPAR agonists have been shown to increase *LRP2* expression in renal tubular cells [29]. Similarly, putative PPAR binding sites have been found in the promoter region of the human *CUBN* gene, with PPAR-alpha agonists enhancing its expression in renal tubular cells [30]. PPAR-alpha agonists also upregulate *FABP1* transcription [31]. Therefore, fatty acids, which serve as natural ligands for PPARs [29], are likely modulators of these three genes. A proteomic study on renal tubular cells revealed high expression of lipid metabolism enzymes transcriptionally regulated by PPAR-alpha/gamma in proximal tubules, whereas distal tubules exhibited elevated glycolytic enzyme expression; these findings highlight active lipid metabolism (and suppressed glycolysis) in proximal renal tubules [32], which may have important implications for the development of renal disease in the context of poorly controlled diabetes, where lipid profile disturbances are common.

The finding that ARB reduced the expression of *LRP2* and *CUBN* is challenging to interpret considering prior research, which proposed that one protective mechanism of ARB involves restoring *LRP2* expression in the proximal tubule. In rats, ARB-induced increases in *LRP2* expression enhanced albumin endocytosis, thereby reducing urinary albumin loss [33]. A more recent study demonstrated that, in cultured proximal tubular cells, high glucose concentrations led to increased angiotensin II production, activation of the angiotensin II Type 1 receptor, and subsequent reduction in LRP2 expression. Treatment with losartan abolished this response, restoring LRP2 expression [34].

However, studies on the role of LRP2 and CUBN in renal disease progression have yielded conflicting results. Tubular activation of the renin–angiotensin system (RAS) is implicated in hypertension, albuminuria, tubular apoptosis, and tubulointerstitial fibrosis. In vitro studies suggest that LRP2 may be involved in the activation of RAS components in the proximal tubule, and its deficiency could be protective against the activation of inflammatory and profibrotic pathways [35]. It has also been demonstrated that albumin can activate the NLRP3 inflammasome, triggering the secretion of IL18 and IL1 beta after interacting with LRP2 and CUBN in renal tubular cells [36]. The positive correlation between *LRP2* and *IL18* observed exclusively in the T1D group suggests a potential link between *LRP2* and NLRP3 inflammasome activation. Since LRP2 activation by albumin can stimulate both the RAS and the NLRP3 inflammasome, the observed reduction in *LRP2* and *CUBN* expression in the urinary sediment of T1D individuals using ARB may indicate a protective effect of this drug class, potentially attenuating these harmful pathways.

Another pathway that activates the NLRP3 inflammasome involves TGF beta, a growth factor stimulated by the biochemical pathways triggered by hyperglycemia, which, in turn, has its signaling exacerbated by the inflammasome [37]. Very strong and moderate positive correlations were observed, exclusively in the T1D group, between *TGFB1* and *IL1B* expressions, as well as between *TGFB1* and *IL18* expressions, respectively. Furthermore, *IL1B* and *TGFB1* expressions exhibited positive correlations with UAE, indicating that gene expression in urinary sediment cells reflects the involvement of these pathways in the pathogenesis of DKD.

The negative, albeit weak, correlations between plasma concentrations of TC, TG, and *FABP1* expression suggest a compensatory mechanism to maintain lipid homeostasis in renal cells. The correlation observed between BMI and *IL18* expression (*r* = 0.24) is comparable to that observed between BMI and plasma IL18 concentrations (*r* = 0.26) in a study with 955 individuals without diabetes which evaluated the association of this cytokine to metabolic syndrome risk factors [38]. Individuals with T1D may be at higher risk of weight gain due to insulin anabolic effects, and obesity has already been linked to a greater risk of reduced eGFR in this population [39]. The positive correlation between BMI and *IL18* expression in renal cells could be one of the mechanisms contributing to worse renal outcomes when obesity is associated with T1D.

Among the limitations of the present study are the relatively small sample size, which is nonetheless justified by the challenge of obtaining high-quality RNA from urinary sediment, and the predominance of women in the sample. This gender imbalance, commonly observed in our studies on T1D, where women typically constitute around 60% of the cohort, was even more pronounced in this study. This overrepresentation of females may limit the generalizability of our findings, given the known sex-related differences in DKD progression [40].

## 5. Conclusions

In urinary sediment cells of T1D individuals, correlations among the expression of genes encoding proteins involved in lipid metabolism with the expression of proinflammatory and profibrotic genes, as well as with plasma FFA and TG, provide indirect evidence for the role of these lipids in the pathogenesis of DKD [41]. Additionally, the expression profile of some genes showed associations with clinical indicators of renal function, pointing to their potential biological relevance.

## Figures and Tables

**Table 1:** Demographic, clinical, and biochemical characteristics of Type 1 diabetes individuals stratified according to the status of renal function decline and of urinary albumin excretion.

	**Nondecliners (** **n** = 64**)**	**Decliners (** **n** = 23**)**	**p**	**U** **A** **E** ≤ 11** mg/g C (n** = 38**)**	**U** **A** **E** > 11** mg/g C (n** = 49**)**	**p** **value**
Sex (female) (%)	78	78	ns	77	79	ns
Age (years)	40 (27–47.7)	29 (24–51)	ns	39 (26–47.8)	33 (26–49.5)	ns
Age at diabetes diagnosis (years)	13 (5.2–19)	11 (7–14)	ns	11.5 (4.2–18.8)	12.5 (7–19.5)	ns
Duration of diabetes (years)	24 (14.5–35)	19 (16–33)	ns	22.5 (16–34)	20.5 (14–35.5)	ns
BMI (kg/m^2^)	25.6 (22.2–28.2)	22.7 (20.7–26.7)	0.080	24.8 (21.6–28.1)	22.7 (20.6–27)	ns
Arterial hypertension (%)	20.3	30.4	ns	12.5	36.8	0.01
Systolic blood pressure (mmHg)	120 (110–124)	110 (100–125)	ns	110 (101–124)	120 (110–130)	ns
Diastolic blood pressure (mmHg)	75 (70–80)	70 (60–80)	ns	71 (70–80)	80 (70–80)	ns
First HbA1c (mmol/mol)	81 (68–93)	86 (74–103)	ns	81 (67–97)	81 (72–91)	ns
First HbA1c (%)	9.6 (8.4–10.7)	10.0 (8.9–11.6)	ns	9.6 (8.3–11.0)	9.6 (8.7–10.5)	ns
Last HbA1c (mmol/mol)	67 (57–78)	74 (66–95)	0.032	67 (56–76)	74 (64–99)	0.009
Last HbA1c (%)	8.3 (7.4–9.3)	8.9 (8.2–10.8)	ns	8.3 (7.3–9.1)	8.9 (8.0–11.2)	0.009
TC (mmol/L)	4.9 (4.1–5.7)	5.4 (3.9–6.8)	ns	4.9 (3.9–5.5)	5.2 (4.2–6.5)	ns
HDL-c (mmol/L)	1.3 (1.0–1.5)	1.4 (1.0–1.7)	ns	1.3 (1.0–1.7)	1.3 (1.1–1.6)	ns
LDL-c (mmol/L)	3.0 (2.2–3.9)	3.4 (2.7–4.2)	ns	2.8 (2.3–3.6)	3.2 (2.2–4.1)	ns
TG (mmol/L)	1.1 (0.8–1.6)	1.4 (1.0–2.3)	0.066	1.0 (0.8–1.5)	1.4 (0.9–1.9)	0.041
FFA (mmol/L)	1.15 (0.67–1.86)	1.18 (0.64–1.80)	ns	0.91 (0.60–1.80)	1.34 (0.82–2.55)	0.075
Time between first and last evaluation (years)	15.5 (9.2–19)	6 (3–10)	< 0.001	15 (6.2–18.8)	10.5 (4.8–17)	ns
First eGFR (mL/min/1.73 m^2^)	118 (103–131)	121 (111–133)	ns	121 (107–134)	114 (93–130)	ns
Last eGFR (mL/min/1.73 m^2^)	107 (90–123)	68 (40–111)	0.003	104 (90–124)	98 (55–119)	ns
UAE (mg/g creatinine)	9 (4.2–18)	54 (6.3–1,848)	0.0008	5 (3.3–8.6)	42.5 (23–370)	< 0.0001
Categories of UAE			0.0001			0.0001
A1 (%)	79.3	43.5		100	31.6	
A2 (%)	17.5	21.7		0	42.1	
A3 (%)	3.2	34.8		0	26.3	
Use of ACEI (%)	26.5	34.8	ns	23	36.8	ns
Use of ARB (%)	14	34.8	0.061	12.5	29	ns
Use of statin (%)	51.5	50.6	ns	41.7	60.5	ns

*Note:* Data are expressed as median and interquartile range. Nondecliners: estimated glomerular filtration rate (eGFR) decline < 3.5 mL/min/1.73 m^2^/year; decliners: eGFR decline ≥ 3.5 mL/min/1.73 m^2^/year; A1: < 30 mg/g creatinine (C); A2: 30–299 mg/g C; A3: > 300 mg/g C.

Abbreviations: ACEI, angiotensin converting enzyme inhibitor; ARB, angiotensin II receptor blocker; BMI, body mass index; FFA, free fatty acid; HDL-c, high-density lipoprotein cholesterol; LDL-c, low-density lipoprotein cholesterol; ns, nonsignificant; TC, total cholesterol; TG, triglycerides; UAE, urinary albumin excretion.

**Table 2:** Gene expression correlations in the Type 1 diabetes (T1D) group and in the control group without diabetes.

	**T1D**		**Control**	
	**r**	**p** **value**	**r**	**p** **value**
*CUBN* x *FABP1*	0.92	< 0.0001	0.84	< 0.0001
*CUBN* x *LRP2*	0.83	< 0.0001	0.82	< 0.0001
***TGFB1* x *IL1B***	0.82	< 0.0001	—	—
*SLC27A1* x *SLC27A4*	0.77	< 0.0001	0.61	0.011
*LRP2* x *FABP1*	0.77	< 0.0001	0.86	< 0.0001
*LRP2* x *SLC27A1*	0.76	< 0.0001	0.70	0.002
*FABP1* x *SLC27A2*	0.75	< 0.0001	0.72	0.002
*LRP2* x *SLC27A4*	0.73	< 0.0001	0.71	0.002
*LRP2* x *SLC27A2*	0.73	< 0.0001	0.85	< 0.0001
*CUBN* x *SLC27A2*	0.73	< 0.0001	0.72	0.002
*SLC27A1* x *SLC27A2*	0.69	< 0.0001	0.68	0.005
*CUBN* x *SLC27A1*	0.66	< 0.0001	0.73	0.001
***SLC27A1* x *CD36***	0.65	< 0.0001		
***SLC27A4* x *TGFB1***	0.61	< 0.0001		
*SLC27A2* x *SLC27A4*	0.60	< 0.0001	0.50	0.050
*SLC27A4* x *IL18*	0.60	< 0.0001	0.54	0.030
*SLC27A1* x *FABP1*	0.59	< 0.0001	0.73	0.001
***TGFB1* x *IL18***	0.58	< 0.0001	—	—
***SLC27A4* x *CUBN***	0.53	< 0.0001		
***SLC27A4* x *CD36***	0.53	< 0.0001		
*LRP2* x *CD36*	0.52	< 0.0001	0.50	0.044
***SLC27A4* x *IL1B***	0.46	< 0.0001		
***CD36* x *IL1B***	0.46	< 0.0001	—	—
***SLC27A1* x *TGFB1***	0.45	< 0.0001		
***CD36* x *TGFB1***	0.45	< 0.0001	—	—
*FABP1* x *SLC27A4*	0.45	< 0.0001	0.52	0.037
***LRP2* x *IL18***	0.44	< 0.0001	—	—
***SLC27A2* x *IL18***	0.43	< 0.0001		
***SLC27A2* x *CD36***	0.42	< 0.0001		
***IL18* x *IL1B***	0.42	< 0.0001	—	—
*CUBN* x *CD36*	0.40	0.0001	0.52	0.040
*FABP1* x *CD36*	0.35	0.0008	0.59	0.014
***SLC27A1* x *IL1B***	0.32	0.002		
*LRP2* x *TGFB1*	**0.30**	**0.005**	—	—
***SLC27A2* x *IL1B***	**0.28**	**0.010**		

*Note:* Correlations in bold were observed only in the group with Type 1 diabetes.

**Table 3:** Correlations among gene expression and other variables in the Type 1 diabetes group.

**UAE x genes**	**r**	**p** **value**
UAE x *IL1B*	0.26	0.016
UAE x *TGFB1*	0.23	0.034

**Plasma lipid concentrations x genes**	**r**	**p** **value**
FFA x *CD36*	0.27	0.025
FFA x *IL1B*	0.30	0.011
TG x *CD36*	0.22	0.046
TG x *FABP1*	−0.28	0.009
TC x *FABP1*	−0.23	0.036

**Other variable x genes**	**r**	**p** **value**
BMI x *IL18*	0.24	0.025

Abbreviations: BMI, body mass index; FFA, free fatty acid; TC, total cholesterol; TG, triglycerides; UAE, urinary albumin excretion.

## Data Availability

The datasets used and/or analyzed during the current study are available from the corresponding author on reasonable request.

## Nomenclature

ACEI: angiotensin converting enzyme inhibitor
ARB: angiotensin II receptor blocker
BMI: body mass index
CD36: cluster differentiation 36
cDNA: complementary DNA
CKD-EPI: Chronic Kidney Disease Epidemiology Collaboration
CUBN: cubilin
DCCT/EDIC: the Diabetes Control and Complications Trial/Epidemiology of Diabetes Interventions and Complications
DKD: diabetic kidney disease
eGFR: estimated glomerular filtration rate
FABP1: fatty acid binding protein 1
FATPs: fatty acid transporter proteins
FFA: free fatty acid
HDL-c: high-density lipoprotein-cholesterol
IL1B: interleukin 1 beta
IL18: interleukin 18
LDL-c: low-density lipoprotein-cholesterol
LRP2: LDL receptor related protein 2 (megalin)
mRNA: messenger ribonucleic acid
NLRP3: nucleotide-binding domain, leucine-rich–containing family, pyrin domain–containing-3
PPAR: peroxisome proliferator-activated receptor
RAS: renin–angiotensin system
SLC27A: solute carrier family 27
T1D: Type 1 diabetes
TC: total cholesterol
TG: triglycerides
TGFB1: transforming growth factor beta 1
TNF: tumor necrosis factor
UAE: urinary albumin excretion
VLDL: very low-density lipoprotein

## Supporting Information

Supporting Information 1 Table S1: Catalog numbers (Assay IDs) for the TaqMan probes and primers used to evaluate gene expression in urinary sediment cells. Table S2: Demographic and clinical characteristics of the control group and of the type 1 diabetes (T1D) group.

Supporting Information 2 Figure S1: Representative image of RNA extracted from urinary sediment cells evaluated in a 2100 Bioanalyzer capillary electrophoresis system (Agilent Technologies, Santa Clara, USA).

Supporting Information 3 Figure S2: Relative expression of the genes *CD36* (A), *FABP1* (B), *SLC27A1* (C), *SLC27A2* (D), *SLC27A4* (E), *LRP2* (F), *CUBN* (G), *IL1B* (H), *IL18* (I), and *TGFB1* (J) in urinary sediment cells from individuals without diabetes mellitus (control group) and with type 1 diabetes mellitus (T1D). The horizontal line within each box plot represents the median, the box plot limits refer to the interquartile range (25th to 75th percentiles), and the bars represent the 10th and 90th percentiles.
