# Supplementary material for: Gene Expression in Urinary Sediment Cells as an Indicator of the Contribution of Plasma Lipids to Diabetic Kidney Disease
Source: J Diabetes Res. 2025 Oct 7;2025:2349928. doi: 10.1155/jdr/2349928 (PMC12520818; doi:10.1155/jdr/2349928)

**Supplementary Material**

**Supplementary Table 1.** Catalog numbers (Assay IDs) for the TaqMan probes and primers used to evaluate gene expression in urinary sediment cells.

| **Gene symbol** | **Gene name** | **TaqMan Assay ID** |
| --- | --- | --- |
| *ELF1* | E74-like factor 1 | Hs00152844_m1 |
| *CD36* | Cluster of differentiation 36 | Hs00354519_m1 |
| *FABP1* | Fatty acid binding protein 1 | Hs00155026_m1 |
| *SLC27A1* | Solute carrier family 27 member 1 (FATP1) | Hs01587917_m1 |
| *SLC27A2* | Solute carrier family 27 member 2 (FATP2) | Hs00186324_m1 |
| *SLC27A4* | Solute carrier family 27 member 4 (FATP4) | Hs00192700_m1 |
| *LRP2* | Low density lipoprotein receptor-related protein 2 (megalin) | Hs00189742_m1 |
| *CUBN* | Cubilin | Hs00153607_m1 |
| *IL1B* | Interleukin 1 beta | Hs01555410_m1 |
| *IL18* | Interleukin 18 | Hs01038788_m1 |
| *TGFB1* | Transforming growth factor beta 1 | Hs00998133_m1 |

**Supplementary Table 2.** Demographic and clinical characteristics of the control group and of the type 1 diabetes (T1D) group.

| **Variable** | **Control group (n=16)** | **T1D group (n=87)** | **p-value** |
| --- | --- | --- | --- |
| Age (years) | 40.0 (29.0–52.5) | 37.5 (26.0–47.75) | ns |
| Sex (female, %) | 68.8 | 77.3 | ns |
| BMI (kg/m²) | 24.0 (22.2–28.7) | 24.8 (21.5–27.8) | ns |
| Arterial hypertension (%) | 12.5 | 22.7 | ns |

Data are expressed as median and interquartile range. BMI: body mass index; ns: non-significant.

**Supplementary Figure 1***.* Representative image of RNA extracted from urinary sediment cells evaluated in a 2100 Bioanalyzer capillary electrophoresis system (Agilent Technologies, Santa Clara, USA).

*
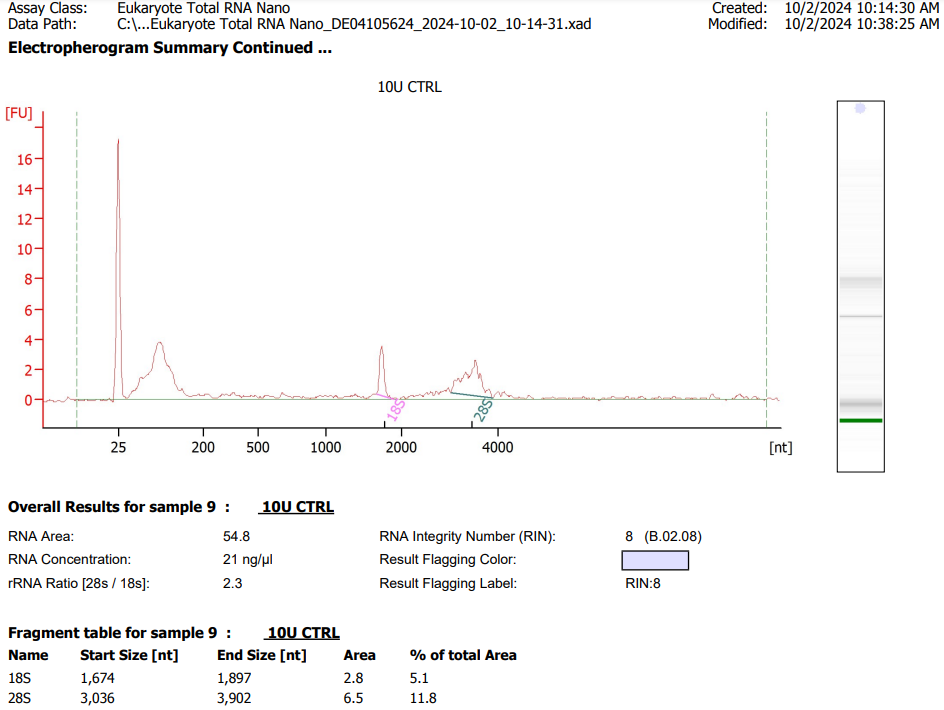
*

**Supplementary Figure 2***.* Relative expression of the genes *CD36* (A), *FABP1* (B), *SLC27A1* (C), *SLC27A2* (D), *SLC27A4* (E), *LRP2* (F), *CUBN* (G), *IL1B* (H), *IL18* (I), and *TGFB1* (J) in urinary sediment cells from individuals without diabetes mellitus (control group) and with type 1 diabetes mellitus (T1D). The horizontal line within each box plot represents the median, the box plot limits refer to the interquartile range (25th to 75th percentiles), and the bars represent the 10th and 90th percentiles.


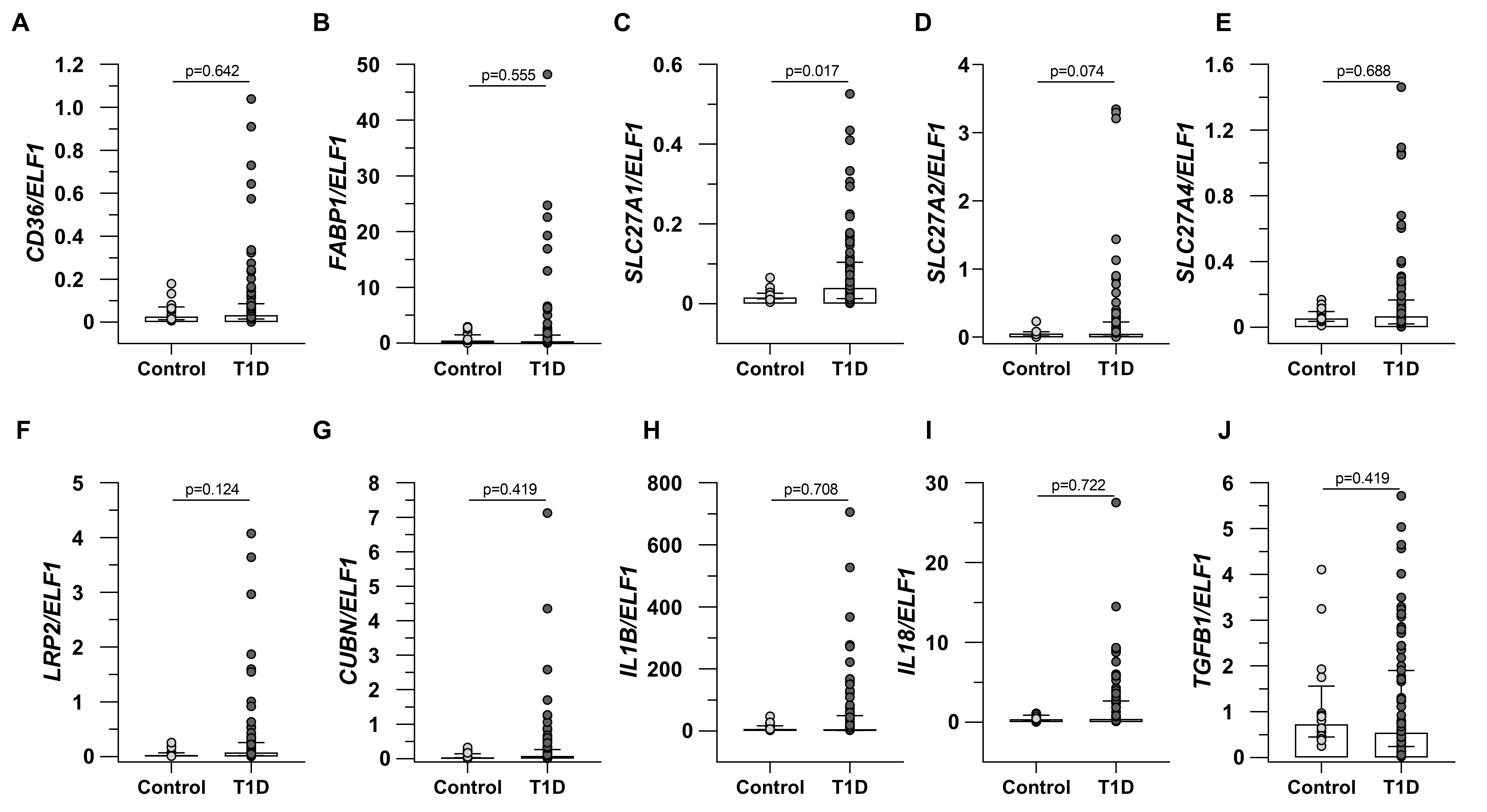

Supplement: Supporting Information 1 — Table S1: Catalog numbers (Assay IDs) for the TaqMan probes and primers used to evaluate gene expression in urinary sediment cells. Table S2: Demographic and clinical characteristics of the control group and of the type 1 diabetes (T1D) group. [file 2349928.f1.docx]
