# Supplementary material for: Gene Expression in Urinary Sediment Cells as an Indicator of the Contribution of Plasma Lipids to Diabetic Kidney Disease
Source: J Diabetes Res. 2025 Oct 7;2025:2349928. doi: 10.1155/jdr/2349928 (PMC12520818; doi:10.1155/jdr/2349928)
Supplement: Supporting Information 2 — Figure S1: Representative image of RNA extracted from urinary sediment cells evaluated in a 2100 Bioanalyzer capillary electrophoresis system (Agilent Technologies, Santa Clara, USA). [file 2349928.f2.pdf]

Electropherogram Summary Continued ...

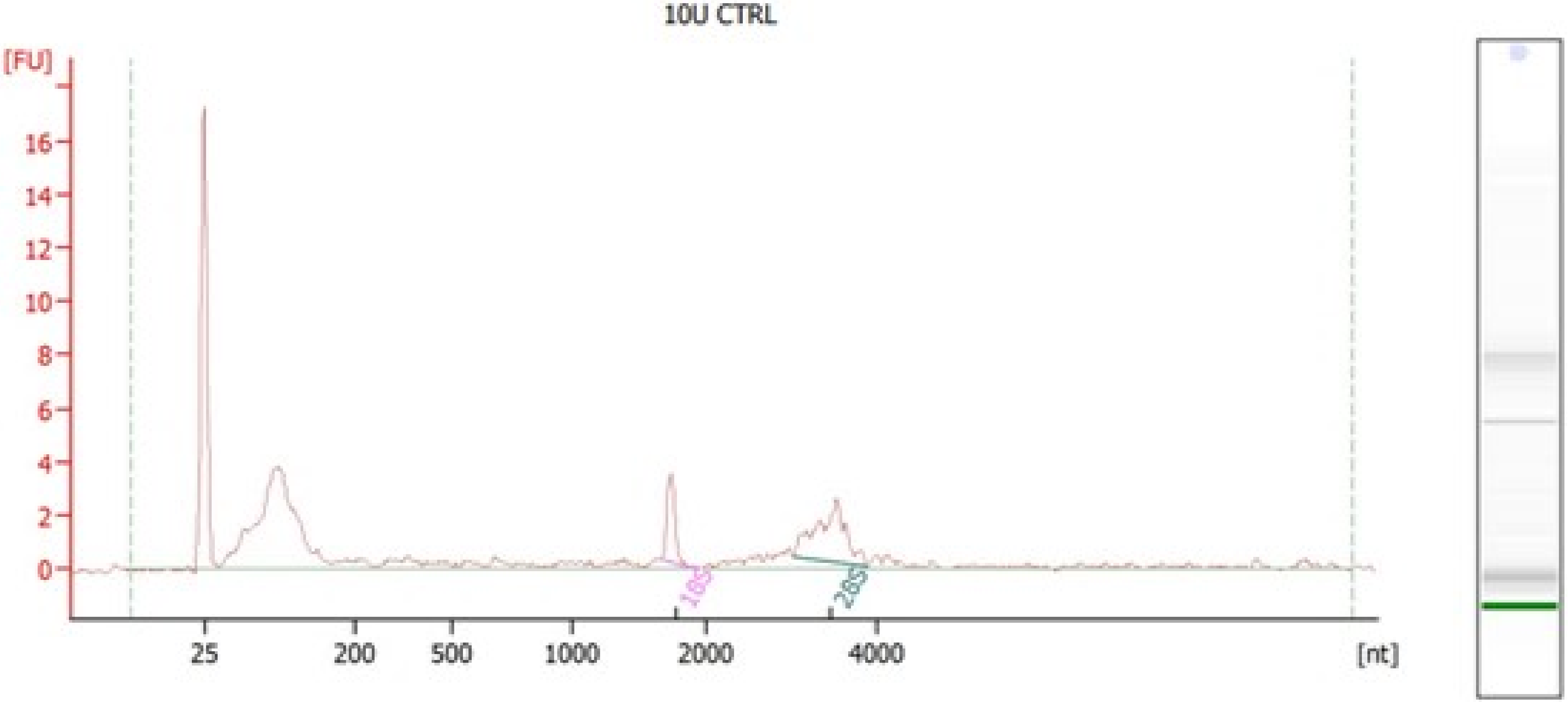

Overall Results for sample 9 : 10U CTRL

RNA Area:	54.8	RNA Integrity Number (RIN):	8 (B.02.08)
RNA Concentration:	21 ng/µl	Result Flagging Color:	
rRNA Ratio [28s / 18s]:	2.3	Result Flagging Label:	RIN:8

Fragment table for sample 9 : 10U CTRL

Name	Start Size [nt]	End Size [nt]	Area	% of total Area
18S	1,674	1,897	2.8	5.1
28S	3,036	3,902	6.5	11.8
