# Supplementary figures and images for: Gene Expression in Urinary Sediment Cells as an Indicator of the Contribution of Plasma Lipids to Diabetic Kidney Disease
Source: J Diabetes Res. 2025 Oct 7;2025:2349928. doi: 10.1155/jdr/2349928 (PMC12520818; doi:10.1155/jdr/2349928)

**A**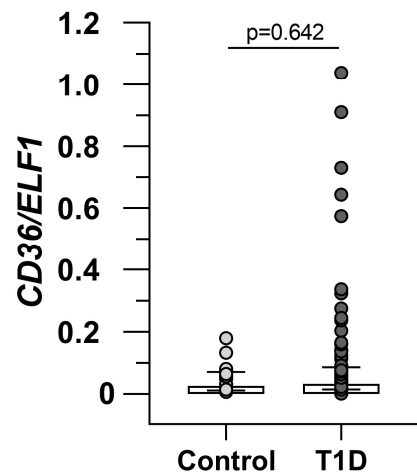**B**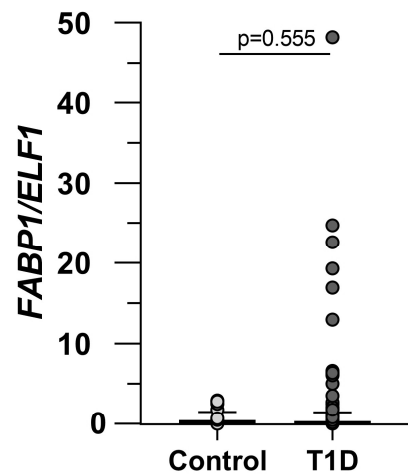**C**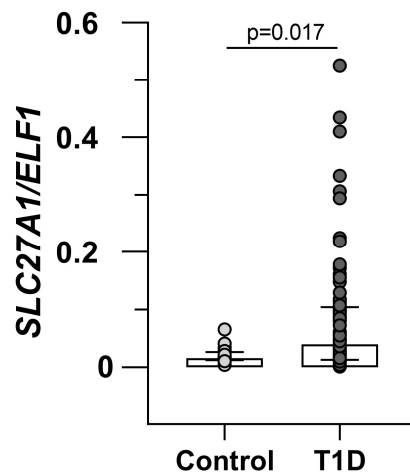**D**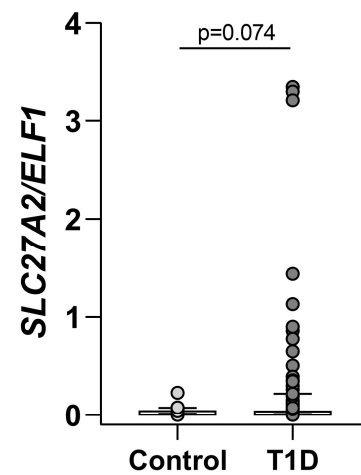**E**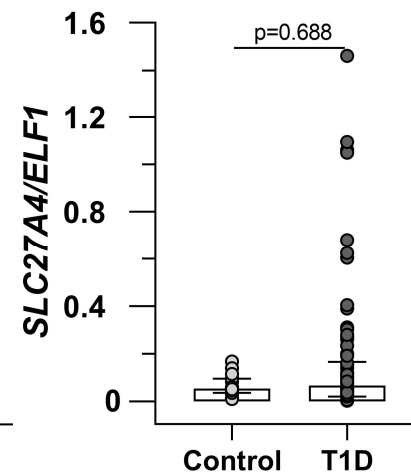**F**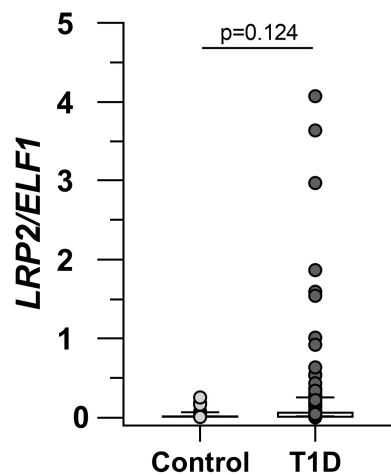**G**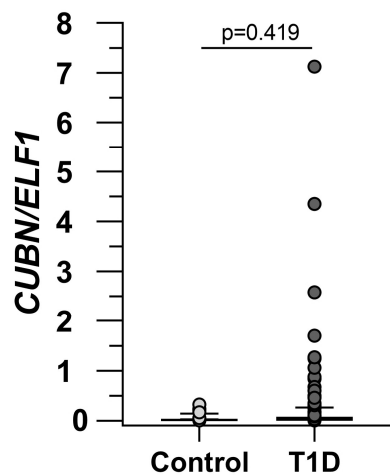**H**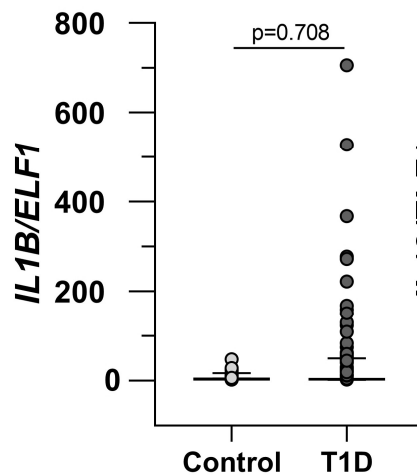**I**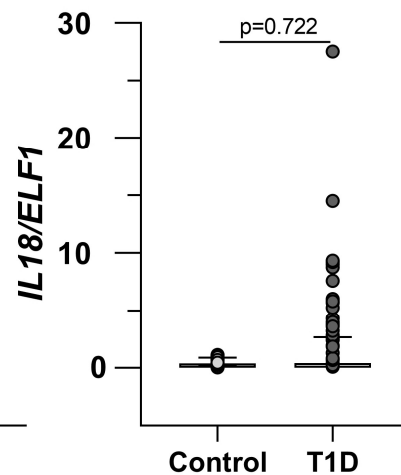**J**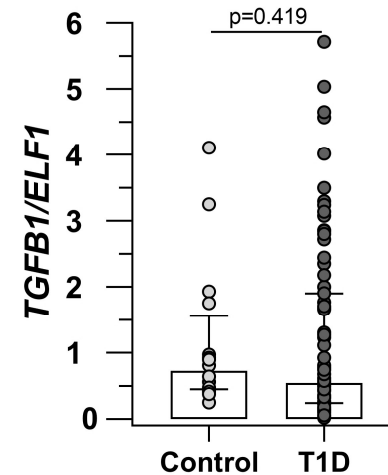

Supplement: Supporting Information 3 — Figure S2: Relative expression of the genes CD36 (A), FABP1 (B), SLC27A1 (C), SLC27A2 (D), SLC27A4 (E), LRP2 (F), CUBN (G), IL1B (H), IL18 (I), and TGFB1 (J) in urinary sediment cells from individuals without diabetes mellitus (control group) and with type 1 diabetes mellitus (T1D). The horizontal line within each box plot represents the median, the box plot limits refer to the interquartile range (25th to 75th percentiles), and the bars represent the 10th and 90th percentiles. [file 2349928.f3.pdf]
